# An evaluation of the 30-s chair stand test in older adults: frailty detection based on kinematic parameters from a single inertial unit

**DOI:** 10.1186/1743-0003-10-86

**Published:** 2013-08-01

**Authors:** Nora Millor, Pablo Lecumberri, Marisol Gómez, Alicia Martínez-Ramírez, Mikel Izquierdo

**Affiliations:** 1 Research, Studies and Sport Medicine Centre, Government of Navarra, Pamplona, Spain; 2 Department of Mathematics, Public University of Navarra, Pamplona, Spain; 3 Department of Health Sciences, Public University of Navarra, Pamplona, Spain

**Keywords:** Inertial units, Frailty syndrome, Kinematic parameters, 30-s chair stand test, Signal analysis

## Abstract

**Background:**

A growing interest in frailty syndrome exists because it is regarded as a major predictor of co-morbidities and mortality in older populations. Nevertheless, frailty assessment has been controversial, particularly when identifying this syndrome in a community setting. Performance tests such as the 30-second chair stand test (30-s CST) are a cornerstone for detecting early declines in functional independence. Additionally, recent advances in body-fixed sensors have enhanced the sensors’ ability to automatically and accurately evaluate kinematic parameters related to a specific movement performance. The purpose of this study is to use this new technology to obtain kinematic parameters that can identify frailty in an aged population through the performance the 30-s CST.

**Methods:**

Eighteen adults with a mean age of 54 years, as well as sixteen pre-frail and thirteen frail patients with mean ages of 78 and 85 years, respectively, performed the 30-s CST while threir trunk movements were measured by a sensor-unit at vertebra L3. Sit-stand-sit cycles were determined using both acceleration and orientation information to detect failed attempts. Movement-related phases (i.e. impulse, stand-up, and sit-down) were differentiated based on seat off and seat on events. Finally, the kinematic parameters of the impulse, stand-up and sit-down phases were obtained to identify potential differences across the three frailty groups.

**Results:**

For the stand-up and sit-down phases, velocity peaks and “modified impulse” parameters clearly differentiated subjects with different frailty levels (p < 0.001). The trunk orientation range during the impulse phase was also able to classify a subject according to his frail syndrome (p < 0.001). Furthermore, these parameters derived from the inertial units (IUs) are sensitive enough to detect frailty differences not registered by the number of completed cycles which is the standard test outcome.

**Conclusions:**

This study shows that IUs can enhance the information gained from tests currently used in clinical practice, such as the 30-s CST. Parameters such as velocity peaks, impulse, and orientation range are able to differentiate between adults and older populations with different frailty levels. This study indicates that early frailty detection could be possible in clinical environments, and the subsequent interventions to correct these disabilities could be prescribed before further degradation occurs.

## Background

Frailty occurs often in people older than 65 years (ranging from 7 to 16.3%), and its prevalence increases with age [1-3]. Frail individuals are at particular risk for poor outcomes such as disability, fall, death and hospitalization from minor stressors [4-7]. The diagnosis of frailty is based on several health domains, including physical impairments (e.g., low gait velocity, fatigue and low grip strength), weight loss, and low physical activity [2]. Despite some vagueness in its definition, clinicians have indicated that early detection is one of the most effective methods for reducing the severity of physical frailty and for improving a patient’s well-being. Functional ability assessments aim to detect mobility impairments such as physical weakness so that early interventions are possible.

The 30-s CST is one of the most important functional evaluation clinical tests because it measures lower body strength and relates it to the most demanding daily life activities (e.g., climbing stairs, getting out of a chair or bath tub or rising from a horizontal position) [8-10]. Low levels of body strength are the primary cause of both balance problems and falls in the elderly population [11,12]. The 30-s CST, similar to tests such as the 5-stands test and the timed up and go test (TUG), is able to differentiate between subjects with different functional levels. However, the 30-s CST is also able to assess the fatigue effect causeg by the number of sit-to-stand repetitions. Indeed, the 30-s CST has been widely used in many studies not only to evaluate functional fitness levels [12-14] but also to monitor training [15-18] and rehabilitation [19,20].

Classically, the 30-s CST consists of manually counting the number of sit-stand-sit cycles completed during the 30 seconds of the test. Since the early 1990s, IUs have been increasingly used to measure kinematic and kinetic parameters [21]. This technology is a non-invasive, portable and economical method to capture accelerations and angular velocities in three orthogonal planes [22]. However, signal analysis is needed to separate out the sit-to-stand (SitTS) and/or stand-to-sit (StandTS) transitions from the entire test duration. Recently, a wide range of studies have positively shown that IUs can furnish accurate kinematic transition-related measures, particularly when a test subject is standing up or sitting down, [21,23-26]. There is no gold standard yet, but this task has typically been achieved [24-26], through the use of thresholds on either the angular velocity [27,28] or the acceleration information [29,30]. However, threshold values are hard to generalize, as they are influenced by noise and by movement artifacts. Thus, peak detection techniques, such as those considered here, seem to perform better [31]. Other authors have preferred to obtain transition durations from the orientation signal of the trunk, which is the angle between the vertical axis and the anterior wall of the subject’s thorax. In this paper, the sinus function is used to soften the signal and the time of postural transition are defined from the previous to the posterior maximum from a minimum point which is the transition indicator [32]. A major difficulty associated with transition detection is the fact that movement patterns depend on the subject’s physical condition. Healthy subjects do not show the same transition indicator as frail subjects, and frail subjects may perform several attempts before completing a valid cycle [33]. Thus, this manuscript uses a novel technique to separate the sit-stand-sit cycles and their phases from the remainder of the signal. First, the vertical position signal is used to clearly differentiate the cycles, and then, transition events are detected using both acceleration and orientation signals to separate the phases, which include “impulse”, “stand-up” and “sit-down”.

Vertical position is the most intuitive indicator of up and down movements and is a good source of information to separate sit-stand-sit cycles within the 30-s CST. Several authors have recognized the value of vertical position data for analyzing SitTS and StadTS transitions [34,35] but details about the derivation of the position signal are lacking. To the best of our knowledge, this is the first study that uses the vertical position signal to separate cycles. While this lack is surprising, it is perhaps because obtaining a position value from acceleration data is made difficult by the inherent drift effect. In the proposed method, vertical position and velocity were obtained from acceleration data and drift-corrected. The IU’s vertical position is a square-like signal that reflects the subject’s vertical movement pattern, which is also very useful for the detection of failed attempts.

Furthermore, the joint use of both acceleration and orientation information enables our algorithm to obtain the duration of SitTS and StandTS transitions irrespective of the subject’s physical condition, overcoming one common problem. The onsets of these movements are based on specific notable events such as acceleration or orientation maximum peaks instead of an empirically determined threshold [29,33].

Finally, all of this information (vertical position, velocity, orientation and acceleration) was used to detect failed attempts in which subjects did not reach the upright position. In the literature, only Van Lummel et al., [36] differentiate between correct SitTS movements and failed attempts. They employed the velocity information instead of the position, but they did not provide any further information about their methodology. Our study illustrates how the position and velocity signals were obtained and validates them against the gold standard provided by a Vicon optical system. This is the first study to analyze the 30-s CST and to obtain kinematic measurements related to the subject’s frailty level.

The present study contains two parts. First, a set of parameters from an IU were evaluated to assess their ability to predict the subjects’ frailty status (frail, pre-frail or healthy) according to the Fried et al. classification, [2]. Specifically, we hypothesized that these body motion-related parameters would enhance an analysis of the current test information (the number of completed cycles). Therefore, in the second part of this study, the aforementioned parameters were further assessed in a situation that highlighted the limited sensitivity of the standard test outcome. Specifically, a subset of pre-frail and healthy subjects performing the same number of cycles was chosen, and the proposed procedure was applied to their kinematic data. The sensitivity of the computed parameters to detect subtle differences was analyzed.

## Methods

### Subjects and protocol

In this experimental study, 47 subjects with different frailty levels were asked to perform the 30-s CST. Specifically, 13 frail subjects (4 males and 9 females, aged 85 ± 5 years, body mass 67.5 ± 8.6 kg, and height 1.54 ± 0.05 m), 16 pre-frail (8 males and 8 females, aged 78 ± 3 years, body mass 71.6 ± 10.5 kg, and height 1.61 ± 0.08 m) and 18 healthy subjects (14 males and 4 females, aged 54 ± 6 years, body mass 75.2 ± 3.4 kg, and height 1.76 ± 0.04 m) volunteered to participate in this study. The frail and pre-frail subjects were selected from the population used for the baseline data of the Toledo Study for Healthy Aging (TSHA) [37]. According to the criteria defined by Fried et al., [2], frailty was determined as the presence of three or more of the following criteria: slowness, weakness, weight loss, exhaustion, and low physical activity. Subjects were classified as pre-frail if one or two criteria were present and as non-frail if no criteria were present. All of the subjects were thoroughly informed about the experimental procedure; the purpose, nature, and possible risks associated with the study; and their right to terminate participation at their discretion. Subsequently, the subjects provided their written informed consent to participate. These experimental procedures were approved by the Institutional Review Committee of the Public University of Navarra and the Department of Health Sciences of the Government of Navarra, according to the Declaration of Helsinki.

The 30-s CST consists of standing up and sitting down from a chair as many times as possible within 30 seconds. A standard chair (with a seat height of 40 cm) without a backrest but with armrests was used. Initially, subjects were seated on the chair with their back in an upright position. They were instructed to look straight forward and to rise after the “1, 2, 3, go” command at their own preferred speed with their arms folded across their chest. All trials were performed using the same chair and with similar ambient conditions. The medical staff who supervised the performance of the test did not participate in analyzing the kinematic data, and they did not have any knowledge about the analysis whatsoever.

As described in the background section, the present study contains two parts. In the first part, all of the subjects from the three frailty groups were evaluated. Everyone was able to finish the test properly. In the second part, a subset of the data from the initial test was considered. A group of seven pre-frail and eight healthy subjects, with a mean number of 17 sit-stand-sit cycles per group and a range of 15–20 were evaluated.

### Instrumentation

An inertial MTx Orientation Tracker (WSENS, Xsens Technologies B.V., Enschede, Netherlands) was attached over the L3 region of the subject’s lumbar spine to provide the kinematic data for each trial. It recorded at a sampling rate of 100 Hz. The L3 position was chosen because of its proximity to the body’s center of mass (CoM) in the standing position. The nine individual MEMS sensors from the MTx provided kinematic data such as the 3D acceleration and the 3D rate of turn (rate gyro). Moreover, the drift-free 3D orientation was also provided by the MTx using Kalman filters and the previously mentioned kinematic data.

Before starting the test, when the subject was sitting on the chair in an upright position, the sensor-fixed reference frame was aligned with the global reference system (X, Y, and Z). This global reference system was defined as the Earth-fixed global reference frame (XYZ), whose Z-axis points vertically upwards, with the X-axis in the lateral direction and the Y-axis in the anterior-posterior direction. The orientation data, consisting of the Euler angles in either XYZ or roll-pitch-yaw order, defined the rotation aligning the global axis to the sensor-fixed reference frame at each time point. The IU provides both the linear acceleration and the rate of turn in its sensor-fixed Cartesian reference frame (xyz). The linear acceleration in the global reference frame can be translated into the global reference frame using the orientation data.

### Data analysis

An automated data analysis procedure was implemented using Matlab 7.11 (MathWorks Inc., Natick, MA, USA) to improve the objectivity and simplicity of the current 30-s CST evaluation. The automated analysis provides an accurate count of the number of repetitions, removing failed attempts as determined by the roll rotation angle (X-orientation) in combination with the Z-acceleration signal, and the derived Z-velocity and Z-position, and the kinematic parameters. The procedure was implemented as a three-stage algorithm:

First, the raw signals were processed to obtain the Z-velocity and Z-position. Specifically, single and double integration of the Z-acceleration was performed. Furthermore, a two-step processing method (a fourth-level polynomial curve adjustment followed by baseline interpolation from local maxima and minima) was chosen to correct the inherent drift effect. A fourth-level polynomial fitting was chosen to accommodate for slow changes in the acceleration bias without incurring in over-fitting. Then, remaining baseline fluctuations were estimated by spline interpolation of local maxima and minima.

**Figure 1:**
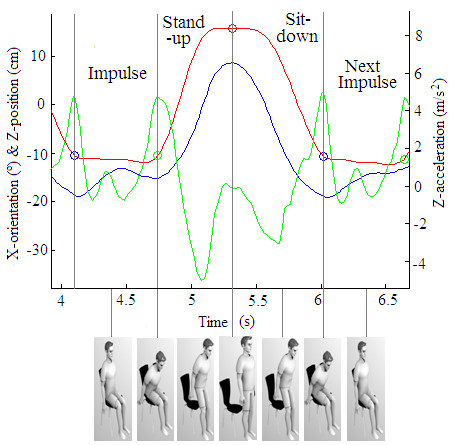
**Raw MTx signals during the 30-s CST of one pre-frail subject.** The blue line is the X-Orientation signal, the green line is the Z-Acceleration signal and the red line is the Z-Position signal from a signal sit-stand-sit cycle. Impulse, stand-up and sit-down phases are also marked.

Second, the corresponding sit-to-stand-to-sit cycles and their main phases (impulse, stand-up and sit-down) [33] were determined using the X-orientation as well as the Z-acceleration, Z-velocity and Z-position. The Z-position signal was used as an indicator of changes in the vertical position of the MTx unit, making it possible to automatically obtain the number of completed cycles (the current standard measurement from the 30-s CST). The X-orientation informs about the body’s sway movements (i.e., forward and backward trunk leans), while the Z-acceleration gives information about the up and down body forces exerted to complete the cycles. The combination of these two signals with the Z-position provides enough markers to clearly detect stand-up and sit-down transitions, as well as failed attempts. A failed cycle was defined as an attempt performed by a subject who did not reach the upright position. In the algorithm, these situations were automatically detected based on a threshold applied to both the time elapsed between a maximum and a minimum of the Z-position and to their difference. The Z-velocity signal was used to establish whether a transition was SitTS or StandTS, (Figure 1), [33].

Finally, to quantify the potential differences between subjects, specific movement-related parameters were derived based on the raw MTx acceleration and orientation signals, as well as additional values obtained after data analysis (i.e., the duration, velocity, and position).

An analysis was performed on the X-orientation and Z-acceleration signals. The X-orientation was selected because it contains information about the way the subjects manage their body (the trunk’s forward and backward tilt), while the Z-acceleration was related to the impulse required to reach the upright position. To evaluate each parameter, the data were first divided into cycles; then, each cycle was separated into its corresponding phases. Therefore, kinematic parameters could be defined in each phase of the performed cycles for any subject. The overall value of a parameter for a subject was obtained by computing its mean value across the subject’s cycles. These parameters describe the subject’s movement performance in terms of the mean, standard deviation, maximum, minimum and range of several features of the subtasks, including the duration of the phases and the orientation, position and acceleration signals. The parameters described below (a, b, and c) were also obtained from our analysis:

### a) X-orientation range

**Figure 2:**
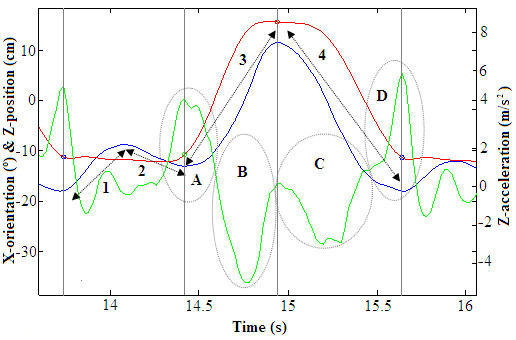
**Explanation of the impulse (A-D) or body management (1–4) parameters.** Numbers 1 to 4 outline the maximum lean backwards and forwards to sit-down and stand up: “TurnB_Sit”, “TurnF_Sit”, “TurnB_Up” and “TurnF_Up”. Capital letters refer to the active and passive impulse to achieve the standing and corresponding sitting positions: “+” and “-“ Up “modified impulses” and “+” and “-“ Down “modified impulses”. Signals are raw MTx ones during the test performed by a pre-frail subject: Z-position (red), Z-acceleration (green) and X-orientation (blue).

Four parameters were defined to characterize the amount of forward and backward trunk tilt occurring during each cycle, (Figure 2, blue line). These parameters were evaluated not according to the phases of the cycles but instead to the trunk movements of the subjects performing SitTS and StandTS transitions. Considering that the cycle starts with the impulse phase, we assumed that the subject was initially in the upright position to define the following ranges of movement:

● TurnB_Sit is a backward trunk lean while the subject is sitting down that is generally produced to accommodate the weight into the chair (Figure 2, blue line, “1”).

● TurnF_Sit is a forward trunk lean in the seated position to start the next standing-up (Figure 2, blue line, “2”).

● TurnB,Up is a backward trunk lean that occurs while the subject is standing-up until he reaches the upright position (Figure 2, blue line, “3”).

● TurnF_Up is a forward trunk lean in the standing position while the subject is descending that is normally generated to improve balance control (Figure 2, blue line, “4”).

### b) Standing-up and sitting-down “modified impulses”

The AUC_Zacc_ parameter was defined as the area under the curve of the acceleration for the duration of the movement (1). It was related to the necessary impulse to stand upright and to return to the seat. As previously defined in [33], the AUC was divided into positive and negative components, according to the direction of the displacement. AUC^+^_Zacc_ referred to the active “modified impulse” used to perform the transition upward, whereas AUC^-^_Zacc_ referred to the passive transition back to the chair (Figure 2).

(1) $$\mathrm{AU}C_{\mathrm{Zacc}}=\int_{t_{i}}^{t_{j}}\;a_{Z}(t)\;\mathrm{dt}$$

### c) Maximum peaks of standing-up and sitting-down velocities

Drift-effect cancellation, which has been previously described, was required to obtain the 3-axis velocity from the corresponding acceleration signals. For simplicity, only the Z-velocity and the Y-velocity were evaluated because these parameters have greater relevance for the transitions. The Z-velocity refers to the vertical movements of each cycle of the 30-s CST, while the Y-velocity is the forward and backward speed when standing-up and sitting-down.

Finally, standard statistical methods were used to calculate the mean and standard deviation (SD) of each phase parameter across both cycles and subjects.

### Statistical analysis

The differences among the three groups (frail, pre-frail and healthy) were determined using a one-way analysis of variance (ANOVA) with Newman-Keuls post-hoc comparisons. When the normality test failed (p < 0.05), the Mann–Whitney rank sum test was employed. A p < 0.01 criterion was used to establish statistical significance. Box plots of each parameter for the different movement phases were used to graphically display the variable’s location. The box itself contained the middle 50% of the data. The upper and lower edges of the box indicated the 75th and 25th percentiles, respectively, and the central line was the median value of the data. The ends of the vertical lines, or “whiskers”, were the minimum and maximum data values, and any points outside the whisker ends represented outliers.

## Results

### Overall 30-s CST outcomes

Healthy subjects performed a significantly (p ≤ 0.001) greater number of sit-to-stand cycles (22 ± 7) during the test duration than did either the pre-frail (15 ± 5) or frail subjects (6 ± 1).

### Time domain analysis

The following list outlines the primary parameters analyzed in the time domain. They are categorized based on the information obtained.

### a) Phase duration

**Figure 3:**
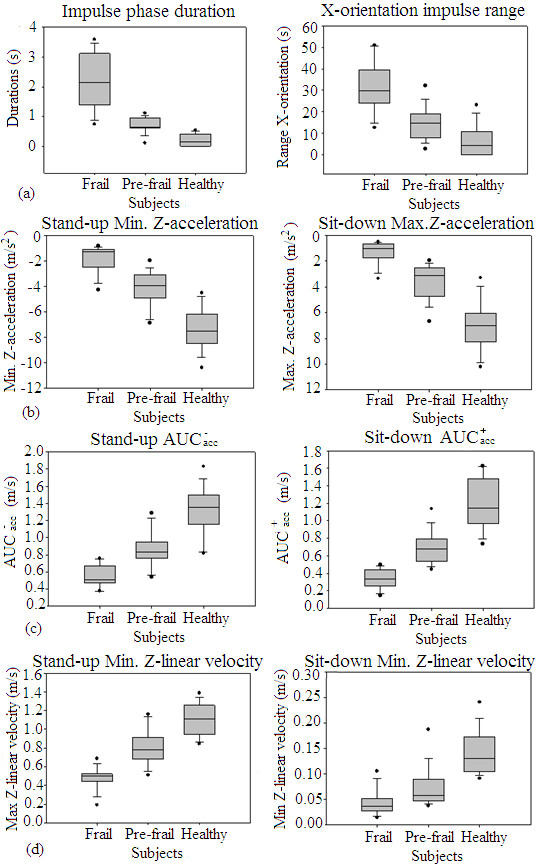
**Box plots of the accelerometer-derived parameters which differentiate between groups for the pairwise comparisons. (a)** represents the time invested for the impulse phase, left side, and the X-orientation range during the impulse phase, right side. **(b)** represents the Z-acceleration minimum values during the stand-up and sit-down and. **(c)** represents the Z-acceleration AUC for the negative and positive impulse when standing-up and sitting-down. Finally, **(d)** represents the maximum and minimum Z-velocity peaks during the stand-up and sit-down.

The duration of the impulse phases was significantly greater (p ≤ 0.001) for frail subjects than for pre-frail and healthy subjects. Other significant differences were also found between pre-frail and healthy subjects (Figure 3a). When the impulse phase duration was normalized to the mean length of the entire cycle, the durations of all phases were significantly smaller (p ≤ 0.001) for the healthy subjects than for pre-frail and frail subjects. However, the differences found between the pre-frail and frail subjects were not significant.

### b) X-orientation

Significant differences were observed among the three groups in the X-orientation range found during the impulse phase (Figure 3a). This value, indicating the subject’s sway, was significantly greater for the frail subjects than for the pre-frail and healthy subjects (p ≤ 0.001).

The frail subjects had a greater X-orientation range during the stand-up phase than did the healthy and pre-frail subjects (p ≤ 0.001), whereas the differences between the ranges of the pre-frail and healthy subjects were not significant.

### c) Linear Z-acceleration

The minimum Z-acceleration values when standing-up and sitting-down were significantly greater for the healthy subjects than for the pre-frail and frail subjects. In addition, these values were significantly (p ≤ 0.001) greater in the pre-frail subjects than in the frail subjects (Figure 3b). However, the maximum Z-acceleration values for the stand-up and sit-down phases only differentiate healthy from frail and pre-frail subjects.

The Z-acceleration “modified impulses” (positive, negative and total) required for the standing-up transition were significantly greater in the healthy subjects than in the pre-frail and frail subgroups. These parameters were also significantly greater for the pre-frail subjects than for the frail (Figure 3c). During the sitting-down transition, the positive impulse was greater in the healthy subjects than in the pre-frail and frail groups. Moreover, this impulse was greater in the pre-frail subjects than in the frail group, while no differences were found between these groups for the negative and total impulses.

### d) Linear Z-velocity

The maximum linear Z-velocity that occurred during the stand-up phase and the minimum linear Z-velocity occurring during the sit-down phase were both significantly greater for the healthy subjects than for the pre-frail and frail groups. Moreover, the pre-frail subjects showed significantly greater values than did those in the frail group (Figure 3d).

The resultant velocity values also indicated similar differences in the maximum values for the standing-up and sitting-down phases.

### Analysis of the pre-frail and healthy subjects with matching cycles

The two groups chosen for their matching cycle numbers were similar with respect to the duration of their phases. Nonetheless, a few of the orientation- and acceleration-derived measures that were able to differentiate frailty groups in the previous analysis could also differentiate the new sub-groups. For example, the maximum Z-velocity during the standing-up phase was greater for the healthy subjects than for the other group, a relationship that was also true for the minimum Z-velocity’s absolute value (Figure 3b). Additionally, the AUC^-^_Zacc_ when standing up and the AUC^+^_Zacc_ when sitting down were significantly greater for the pre-frail subjects than for the healthy subjects (p ≤ 0.001) (Figure 3c).

## Discussion

Our analysis revealed that movement-related parameters such as the phase duration and the angular rate, as well as power-related magnitudes such as acceleration, velocity and AUC_Zacc_, allow us to clearly differentiate between people belonging to different frailty groups (frail, pre-frail and healthy) (Table 1). Moreover, the velocity and AUC_Zacc_ parameters are sensitive enough to differentiate between a pre-frail and a healthy subject when the actual test outcome (number of cycles completed) could not. These results are a preliminary step toward the development of a user-friendly, simple automated tool to help clinicians assess the 30-s CST in an objective manner based on movement-related parameters.

**Table 1:** Evaluated parameters that differentiate frailty levels

**PARAMETERS**	**Frail vs Pre-Frail**	**Frail vs Healthy**	**Pre-Frail vs Healthy**	**Cycles-matched**
Cycles number	YES	YES	YES	NO
IMPULSE PHASE				
Duration	YES	YES	YES	NO
Normalized duration	NO	YES	YES	NO
X-orientation range	YES	YES	YES	NO
STAND-UP PHASE				
X-orientation range	YES	YES	NO	YES
Minimum peak Z-acc.	YES	YES	YES	NO
Maximum Z-acc. peak	NO	YES	YES	NO
AUC_Zacc_	YES	YES	YES	NO
AUC^+^_Zacc_	YES	YES	YES	NO
AUC^-^_Zacc_	YES	YES	YES	YES
AUC_Zacc_	NO	YES	YES	NO
Maximum Z-velocity peak	YES	YES	YES	YES
Maximum Z-velocity peak	YES	YES	YES	YES
SIT-TO-STAND PHASE				
Minimum Z-acc. peak	YES	YES	YES	NO
Maximum Z-acc. peak	NO	YES	YES	NO
AUC^+^_Zacc_	YES	YES	YES	YES
AUC^-^_Zacc_	NO	YES	YES	NO

### Added information from the instrumented 30-s CST

Our hypothesis was that subjects with different frailty levels not only differ in the number of the performed cycles during the 30-s CST but also in their movement pattern, which is constrained by their functional capacity. IUs were presented as a suitable tool to evaluate each cycle and its constituent phases (impulse, stand-up, and sit-down) and from these IUs, the corresponding kinematic information could be obtained.

**Figure 4:**
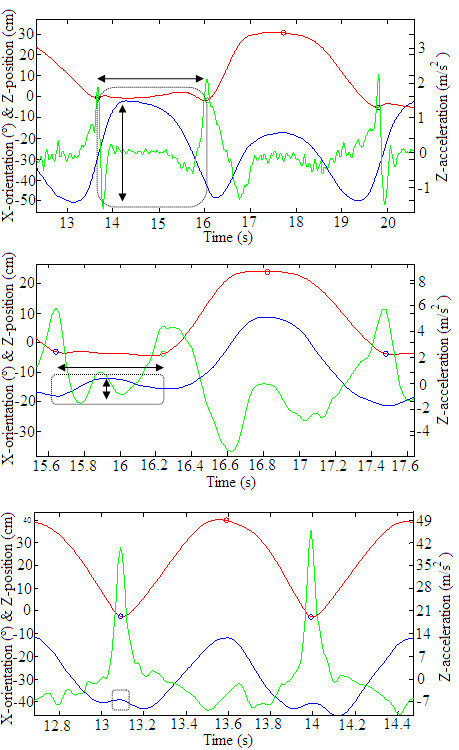
**Movement patterns of raw MTx signals (Z-position, X-orientation, Z-acceleration) for frail (a), pre-frail (b), and healthy subjects (c).** The circle outlines the extra forward and backward lean for more frail subjects and the arrows features the time duration and X-orientation range.

As Ganea et al. showed in a previous study [25], frailty was related to an increased duration of the impulse phase as well as a greater X-orientation range during this phase. This idea is consistent with our results, as it indicates that frail subjects require extra forward and backward leaning to connect one cycle with the next (Figure 4). Moreover, Gross et al. [28] suggested that this extra movement may result from arranging the body to enhance stability or to meet the strength demands of the task. Thus, our hypothesis that different frailty levels lead to different methods of performing movements was corroborated.

Other studies define consecutive sit-stand-sit cycles by a double flexion and extension movement [26,39,40]. Our research showed that this specific movement does not always occur, the X-orientation range we found during the impulse phase revealed that healthy subjects do not perform double flexion and extension. Rather, they are healthy and strong enough to produce the power output with only their lower muscles, so there is no need for any compensatory movement or control strategy. Furthermore, the range of X-orientations during the stand-up and sit-down phases of the frail subjects differed from those of the pre-frail and healthy subjects. Indeed, a frail subject used increased range when leaning backward to sit down and when leaning forward to stand up. No differences in this value were found between healthy and pre-frail subjects.

Frail subjects obtained lower velocity and acceleration peaks as well as reduced impulses (AUC_Zacc_) than did the pre-frail and healthy groups during the stand-up and sit-down phases. Therefore, this frailty group has less physical capacity to perform the different phases of the cycles in the 30-s CST. This finding might be explained by the fact that frail subjects have reduced power in their lower extremities, leading to a restricted and cautious strategy for transition performance [28,41].

Another interesting and promising result was that our method is sensitive enough to detect different frailty levels among groups of subjects with the same number of performed cycles. This finding is especially important because it reveals that other parameters related to the 30-s CST are more sensitive to frailty. In particular, the maximum linear Z-velocity peak during the stand-up phase and the minimum during the sit-down phase, appear to be highly related to frailty. Moreover, the negative “modified impulse”, AUC^-^_Zacc_, that occurs when standing-up (related to the negative acceleration required to reach the upright position), as well as the positive “modified impulse”, AUC^+^_Zacc_, that occurs when sitting down (related to the positive acceleration required to reach the seat), can differentiate pre-frail subjects from healthy ones. This idea could lead to an accurate definition of the frailty syndrome that considers parameters obtained from an IU and is directly related to the movement performance.

In summary, differences in the frailty level of a subject can be found by evaluating the specific way the 30-s CST cycles are carried out. This study highlights the additional backward and forward lean that is produced during the impulse phase for frail subjects, as well as the differences in the forces required to achieve the upward position and to subsequently return to the chair (i.e., decreased Z-acceleration peaks and AUC_Zacc_). Furthermore, the velocities seen in the different phases of each cycle, particularly the Z-velocity peaks of the stand-up and sit-down phases are of special interest because they can differentiate subjects with different levels of frailty that performed the same number of cycles during the 30-s CST.

The present findings motivate future investigations into these topics. For instance, an additional assessment is required to explore the spectral edge frequency information found in the phases of each cycle of the 30-s CST. This manuscript shows that using IUs in the 30-s CST provides kinematic information while maintaining test simplicity and requiring no additional time for data acquisition. Until now, measurements were based on the quantity of cycles, and there was no information about the quality of the movement performance. Furthermore, there is currently no gold standard based on IU data for the assessment of the SitTS and StandTS transitions. The automated approach described in this study may improve doctors’ ability to detect high-risk frailty levels, provide a finer scale for frailty levels, document progression of aging in the elderly, and assess subject responses to exercise programs or other types of interventions in an objective and sensitive manner based on kinematic parameters. However, further research is required to evaluate the association between kinematic 30-s CST variables and other age-related processes in a larger group of subjects.

## Conclusion

The aim of this work is to improve the information gathered with the 30-s CST by defining kinematic parameters that can be gathered from IU data. These parameters could lead to a more precise and reliable determination of a subject’s frailty level (frail, pre-frail, or healthy). This study provides evidence that some of the proposed parameters are more sensitive to the frailty level than the current standard 30-s CST outcome of completed cycles. Positive Z-velocity peaks during the stand-up and sit-down phases, as well as the so-called “modified impulses” stand out as the most promising parameters for the classification task. Therefore, this work offers clinicians a possible method for the early detection of frailty syndrome so that an appropriate treatment can be applied to avoid further decline.

## Abbreviations

CST: Chair Stand Test; IU: Inertial Unit; TUG: Timed Up and Go; TSHA: Toledo Study for Health Aging; CoM: Center of Mass; SitTS: Sit-to-stand; StandTS: Stand-to-sit; AUCZacc: Area Under the Curve of the Z-acceleration; AUC+Zacc: Positive part of the area under the curve of the Z-acceleration; AUC-Zacc: Negative part of the area under the curve or the Z-acceleration; SD: Standard Deviation.

## Competing interests

The authors declare that they have no competing interests.

## Authors’ contributions

NM designed the signal processing, collected, analyzed and interpreted the data, and wrote the article. PL and MG made contributions to the methodology and data interpretation and carefully reviewed the manuscript. AMR assisted with carrying out the experiments, collecting the data, and analyzing the data. MI conceived and designed the study, recruited and consented participants supervised the experiments and performed all the measurements. All authors revised critically the article for important intellectual content and gave the final approval of the version to be published. All authors read and approved the final manuscript.
